# Damage mechanism and therapy progress of the blood-brain barrier after ischemic stroke

**DOI:** 10.1186/s13578-023-01126-z

**Published:** 2023-11-01

**Authors:** Hui-min Gao, Hao Chen, Gui-Yun Cui, Jin-Xia Hu

**Affiliations:** 1 Institute of Stroke Research, Xuzhou Medical University, Jiangsu, China; 2 Department of Neurology, The Affiliated Hospital of Xuzhou Medical University, Jiangsu, China; 3 School of Chemical Engineering and Technology, China University of Mining and Technology, Xuzhou, 221116 China

**Keywords:** Ischemic stroke, Blood-brain barrier, Stroke mechanism

## Abstract

The blood-brain barrier (BBB) serves as a defensive line protecting the central nervous system, while also maintaining micro-environment homeostasis and inhibiting harmful materials from the peripheral blood. However, the BBB’s unique physiological functions and properties make drug delivery challenging for patients with central nervous system diseases. In this article, we briefly describe the cell structure basis and mechanism of action of the BBB, as well as related functional proteins involved. Additionally, we discuss the various mechanisms of BBB damage following the onset of an ischemic stroke, and lastly, we mention several therapeutic strategies accounting for impairment mechanisms. We hope to provide innovative ideas for drug delivery research via the BBB.

## Introduction

Paul Ehrlich [1] made the initial discovery that the dyestuff, when injected into the blood vessels, did not color brain parenchyma. Later, his student Edwin Goldmann [2] made additional observations that showed that the same dyestuff would stain brain tissue if injected into the cerebrospinal fluid. This led to the development of the vague concept of biological barriers between the blood and the brain parenchyma [3]. After conducting extensive research, Dr. Lena Stern presented the term “Barrière hématoencéphalique” to the faculty of Medicine in Geneva [4]. Furthermore, she has published a conceptual article on the topic of the BBB. Following the concrete conceptualization of the BBB, which was developed by Paul Ehrlich, Edwin Goldmann, and Lena Stern, extensive and ongoing studies have been conducted on its morphology, molecular composition, and physiological properties [5]. The BBB maintains the steady-state of the neural system and is therefore considered to be an important structure. Numerous studies have investigated the function of the BBB. Ischemic stroke is a common cause of death worldwide. With the aging of the population, the global burden of stroke is expected to increase [6]. When ischemic stroke occurs, the BBB can be disrupted, resulting in a number of impairments that can worsen the disease’s impact. Currently, maintaining the integrity of the BBB is regarded as an effective treatment strategy for stroke [7].

## Blood-brain barrier

### Composition of BBB

**Fig. 1:**
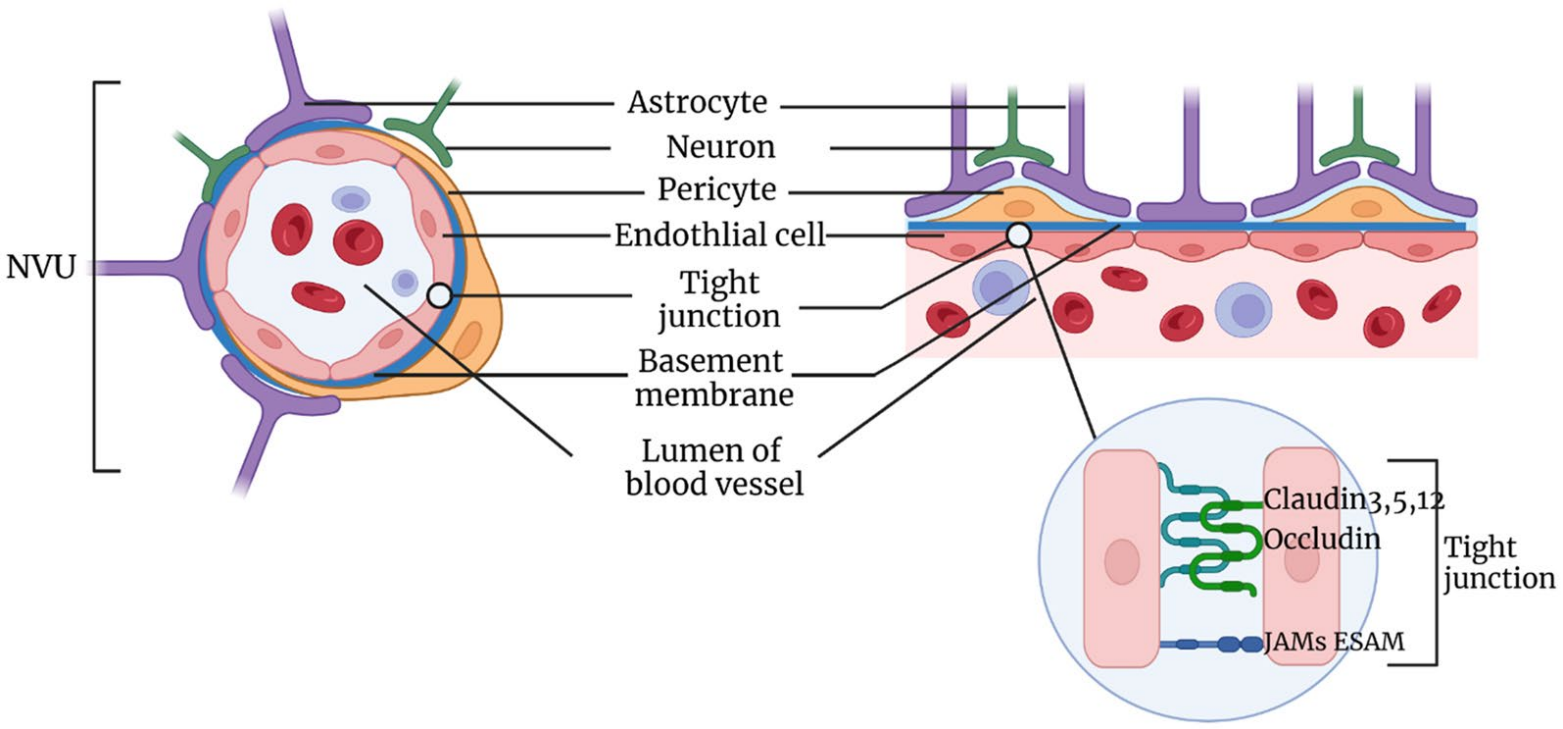
BBB structure

 The neurovascular unit (NVU) serves as the anatomical basis for the BBB (Fig. 1). It is a tightly functioning cellular system consisting of endothelial cells, pericytes and astrocytes. In addition, it receives support from other types of central nervous system structures [8]. The growth and maintenance of BBB are controlled by the interaction between non-cellular elements and endothelial cells [9].

### Neurovascular unit

The neurovascular unit (NVU) [10] is composed of a complex cellular structure, with each component having an intimate relationship that forms a highly efficient system for regulating cerebral blood flow [11]. The BBB is part of the NVU, which exists as a complex that combines vessels and astrocytes with neurons [12]. The NVU typically comprises encephalic vessels, emphasizing the close physical and functional connectivity between brain tissue and vessels [13].

### Endothelial cells and tight junction (TJ)

Endothelial cells belong to the squamous cell family and are vital components that participate in forming the lining of blood vessels. Aside from their function in constructing tight junctions, endothelial cells also express specific transport proteins that regulate the dynamic flow of substrates. More importantly, endothelial cells can manage the transportation of leukocyte cell adhesion molecules to limit inflammatory invasion [14]. The TJ complex consists of transmembrane adhesive proteins that mediate intracellular signal transduction and provide necessary physical support by interacting with corresponding material on the adjacent cytoplasmic membrane [15].

### Pericyte

Pericytes are smooth muscle-like cells that are distributed along capillaries and minute vessels. They possess contractility and are coated within the basement membrane, which plays an important role in maintaining nervous system function. During the period of angiogenesis and maturation of the BBB, pericytes regulate capillary vessel diameter, brain flow [16], and prevent immune cells from penetrating into the central nervous system [9, 17].

### Astrocyte

In the central nervous system, astrocytes have various functions [18]. Their different polarization types possess diverse biochemistry and feature characteristics [19]. Astrocytes can offer neurons energy substrate [20], regulate local blood flow [21], help with drainage of interstitial fluid [22], hold synapse growth and plasticity [23], express benefits on function and behavior about neural circuits [24], and keep the balance state of extracellular fluid,ions, and neurotransmitters [25].

### Microglia cell

Microglia cells are a type of cerebral congenital immune cell [26]. They can adjust tissue development, maintain neural environment stability, promote nerve-repairing procedures, and respond promptly to stressors [27]. Recently, much research indicates that activation of microglia cells is a critical factor in a variety of disease conditions, including ischemic stroke and Alzheimer’s disease [28, 29].

### Extracellular matrix

The extracellular matrix (ECM) is a layer of glycocalyx made up of proteoglycans and glycosaminoglycans that covers the BBB [30]. It functions as the first line of defense against substances attempting to cross the BBB [31]. The ECM proteins and receptors play a crucial role in various molecular signal transductions. They regulate cellular survival, development, metastasis, and differentiation, and enable the brain to adapt to environmental changes [32, 33]. The ECM exists in all tissues and undergoes constant remodeling in response to control signals [33, 34]. Its dynamic structure allows it to regulate cellular signal transduction through integrins and other surface receptors. The ECM’s relative elasticity and ability to sense mechanical signals help regulate organogenesis and adjust cellular metabolism [35]. Matrix metalloproteinases (MMPs) are members of the zinc-dependent endopeptidase family that play an important role in regulating the duration of ECM degradation and remodeling [36]. The expression level and functional changes of MMPs could trigger abnormal ECM degeneration, and are considered an initial signal of disease development [37, 38]. In some pathological conditions, ECM remodeling can become out of control, leading to conditions such as cancer. Excessive accumulation of ECM components is strongly associated with fibrosis [39]. When the BBB is destroyed, plasma protein leakage increases, causing protein retention and leading to adverse effects on the nervous system [40]. The precision of ECM manipulation stems from its composition of glycoproteins, which allows for the use of proteolytic cleavage to modulate the pathophysiological function of the BBB [41]. Currently, numerous methods and drugs are under investigation to modify, modulate, or mimic ECM components in order to effectively treat associated diseases. These efforts demonstrate a commitment to advancing medical research and developing innovative solutions for improved patient outcomes.

### Biological function of the BBB

The BBB is a physical and biochemical barrier that precisely regulates environmental homeostasis in brain tissue. It provides an optimal microenvironment for neurons to perform their functions [42]. Under normal physiological conditions, the BBB ensures a constant supply of nutrients (such as oxygen and glucose) to cerebral cells and directs inflammatory cells to respond to local environmental changes [43–45]. The BBB regulates the microenvironment through its ion channels and transport proteins, maintaining the ion homeostasis and nutrients required by the brain. It also regulates the level of neurotransmitters in the brain and restricts the infiltration of plasma proteins and neurotoxins into the brain [46]. However, if the integrity of the BBB is compromised, it can result in ion imbalance, changes in signal molecule homeostasis, and attack by immune cells and molecules on the central nervous system, leading to neuronal dysfunction, degeneration, and various neurological diseases [47].

### The TJ and junctional proteins of BBB

The TJ [48] primarily consists of occluding proteins and claudins. Among these, claudin-5 is recognized as the dominant TJ protein, making a significant contribution [49]. The zonula occludens (ZO) protein family, a group of membrane-associated guanylate kinase tight junctions, plays a central role in scaffold protein binding to the cytoskeleton [50]. Tight junctions significantly reduce the osmosis of polar solutes from plasma to extracellular fluid through the paracellular pathway [51]. Adhesion junctions [48] involve cadherin, platelet endothelial cell adhesion molecules, and JAM molecules of the adhesion family. Members of this family play an essential role in leukocyte adhesion and trans-BBB migration to the brain parenchyma [52]. However, the specific role of various binding complexes and related proteins in the development and physiological and pathological mechanisms of the BBB requires further clarification. Brain endothelial cells also have gap junctions formed by the connexin family [53], which allow intercellular communication and maintain the integrity of tight connections [48, 54].

### The permeability and pathways through the BBB

The permeability of the BBB is determined by its structure, which is related to the interaction between molecular characteristics and transport proteins. The molecular characteristics that contribute to permeability include molecular weight, hydrogen bonding, polar surface area, electric charge, lipophilicity, and other factors [55–57].

**Fig. 2:**
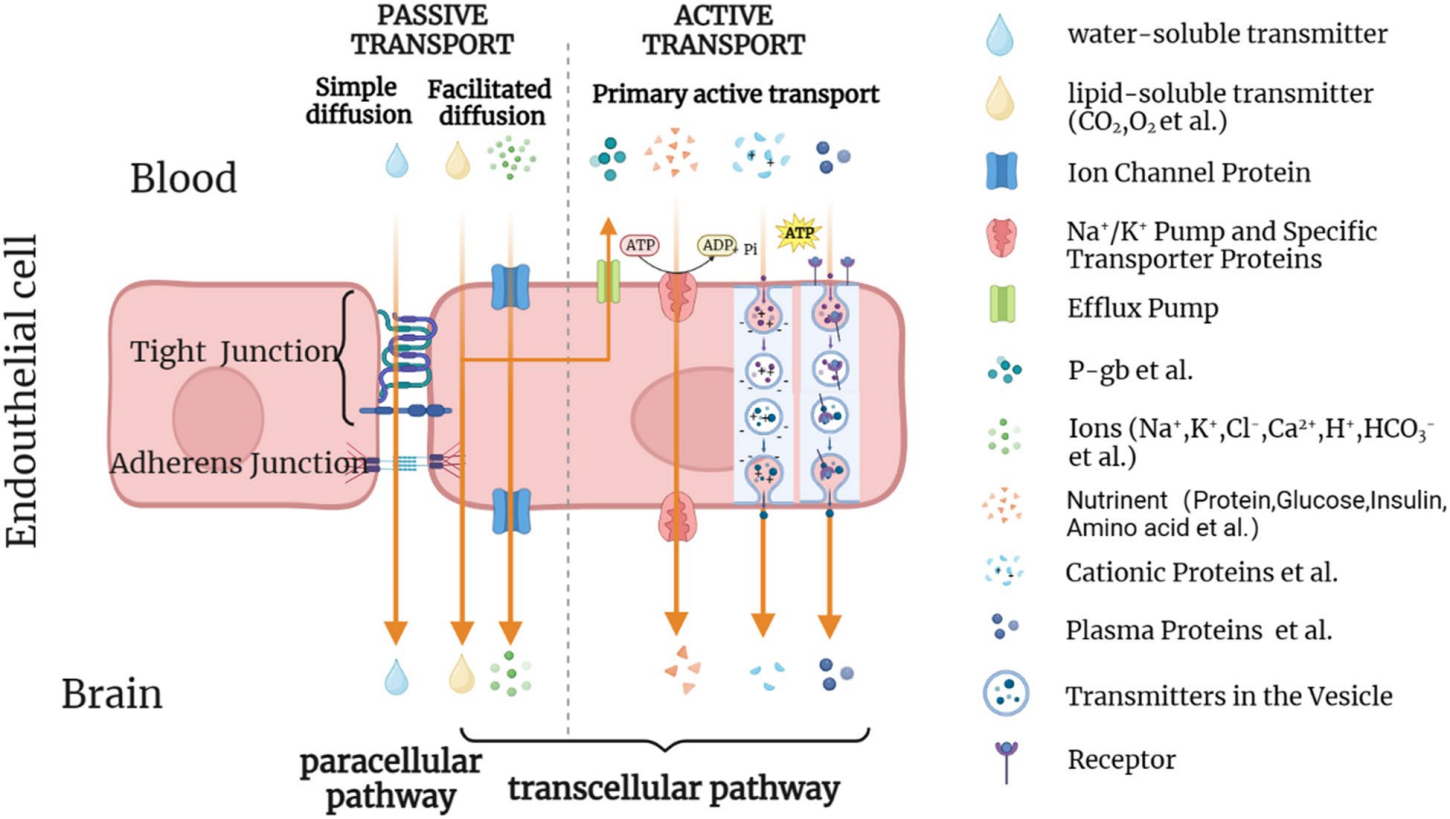
Transportation of substance on the BBB

 There are two ways for materials to traverse the BBB: the transcellular pathway and the paracellular pathway, or, to put it more simply, across the cell itself or via paracellular space at the cell junctions (Fig. 2) [58]. The paracellular pathway is a passive transport mechanism regulated by osmotic pressure and concentration gradient. This process is highly restricted by the TJ and is limited to the diffusion of ions and small hydrophilic molecules [59, 60]. The transcellular pathway is mediated by various mechanisms and is divided into active and passive transport pathways depending on whether energy is consumed during transportation. Passive transport occurs through transcellular diffusion, while the active transcellular transport pathway includes receptor-mediated endocytosis, active efflux transport, and adsorption-mediated endocytosis [61]. Certain gases, like O2 and CO2, can passively diffuse across cells, as can small lipophilic molecules with Log P < 5 and molecular weight < 500 Da [59, 62]. However, polar macromolecules such as peptides and proteins require receptor-mediated endocytosis for transport to their target locations and belong to the active transcellular transport pathway [56, 63, 64]. The BBB expresses various transport proteins, including glucose, choline, and iron transporters, as well as insulin binding proteins and active efflux pumps composed of ATP-binding box transporters [65, 66]. ATP-binding box transporters, like p-glycoprotein, use ATP energy to prevent drugs, exogenous substances, neurotoxic substances, and nucleosides from actively flowing out of endothelial cells into the blood [54]. The transcellular transport pathway is more likely to cross the BBB than the paracellular pathway and is the focus of research for many drug delivery strategies across the BBB [60]. Adsorptive transcytosis relies on the non-specific transport of positively charged substrates, such as cationic bovine serum albumin, interacting with the negatively charged surfaces of brain endothelial cells [67]. The integrity of the BBB is closely associated with the endothelial cell under physiological conditions; but it can be influenced by immune cells, such as microglia and macrophages, during pathological events [68]. Because pathological factors and physical and chemical stimuli can alter the BBB’s permeability, numerous imaging technologies have been developed and applied to measure and evaluate changes in the BBB [63].

### BBB injuries after ischemic stroke

**Fig. 3:**
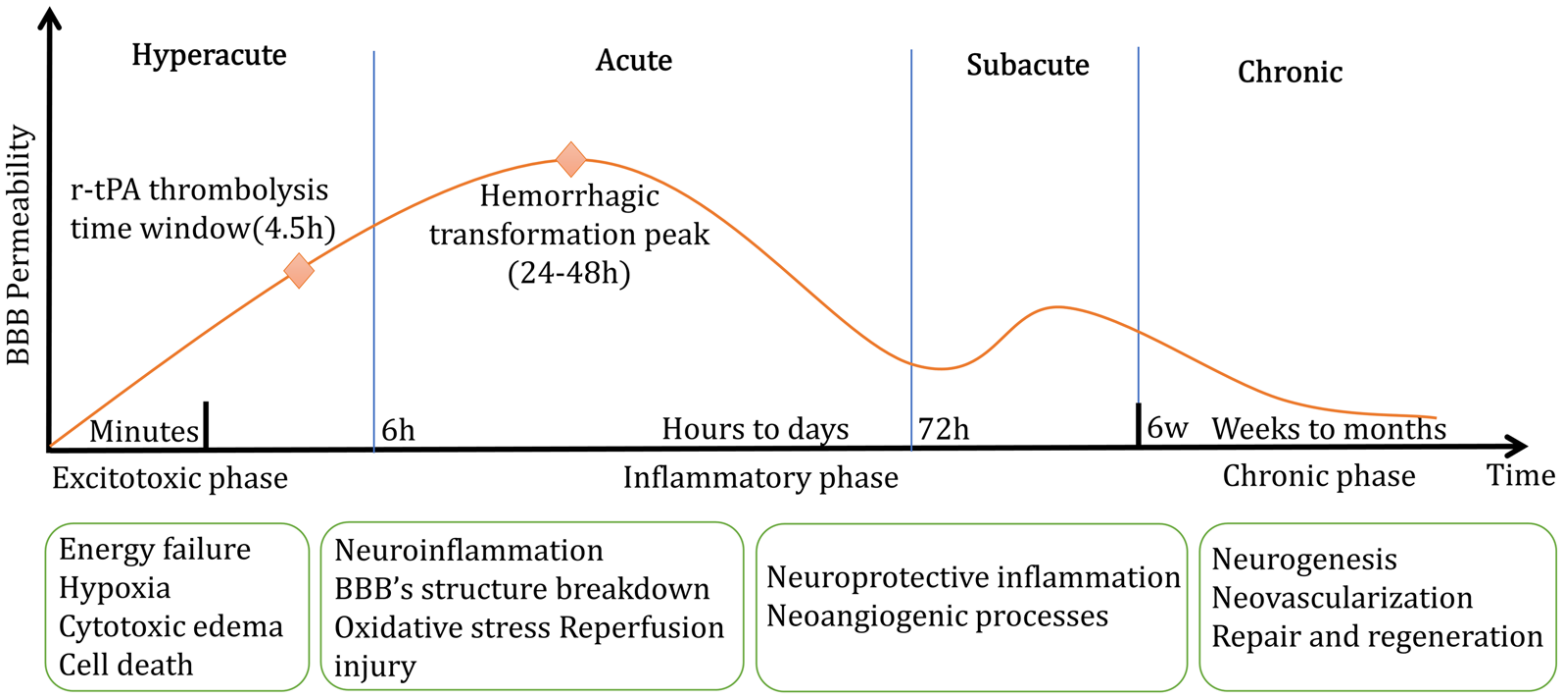
General pathophysiological phases and its main pathophysiological processes of ischemic stroke

 The damage to the BBB is a crucial pathological process of ischemic stroke, which begins during the ischemic phase, worsens during the reperfusion stage, and ultimately results in vasogenic edema and hemorrhagic transformation (Fig. 3) [69–71]. Early-stage pathophysiological events associated with BBB breakdown after an ischemic stroke include the stimulation of sodium transporters (such as Na-K-Cl cotransporters, Na-H exchangers, etc.), which results in edema, and oxidative stress involving reactive oxygen species [72]. The degradation of integrin or TJ proteins also leads to an increase in paracellular leakage of the BBB [43]. Consistent damage to the BBB can cause neuroinflammation and the infiltration and accumulation of immune cells in the brain parenchyma [43].

Ischemic edema is a significant manifestation of ischemic stroke. The reasons for edema are changes in BBB permeability induced by ischemia that result in a series of cytotoxic, ionic, and vasogenic edema events [73–75]. The cell plasma membrane exhibits increased selective permeability, Na+/K+-ATPase, and Ca2+-ATPase activity, leading to the accumulation of sodium-dominated ions and water in cells as a precursor of ionic edema [73, 76, 77]. Many transport proteins close to the BBB may interfere with the stability of the adjustment mechanism on ion channel-associated transporters (Na-H exchangers, Na-Ca exchangers, Na-K-Cl cotransporters), which are non-selective cation channels controlled by sulfonylurea receptor-1 [78]. Vasogenic edema develops from ionic edema. The gaps in the capillaries’ basement membrane widen further due to the breakdown of tight connections between endothelial cells, allowing protein-rich fluid to seep from the extracellular fluid of the brain cells [73, 75].

**Fig. 4:**
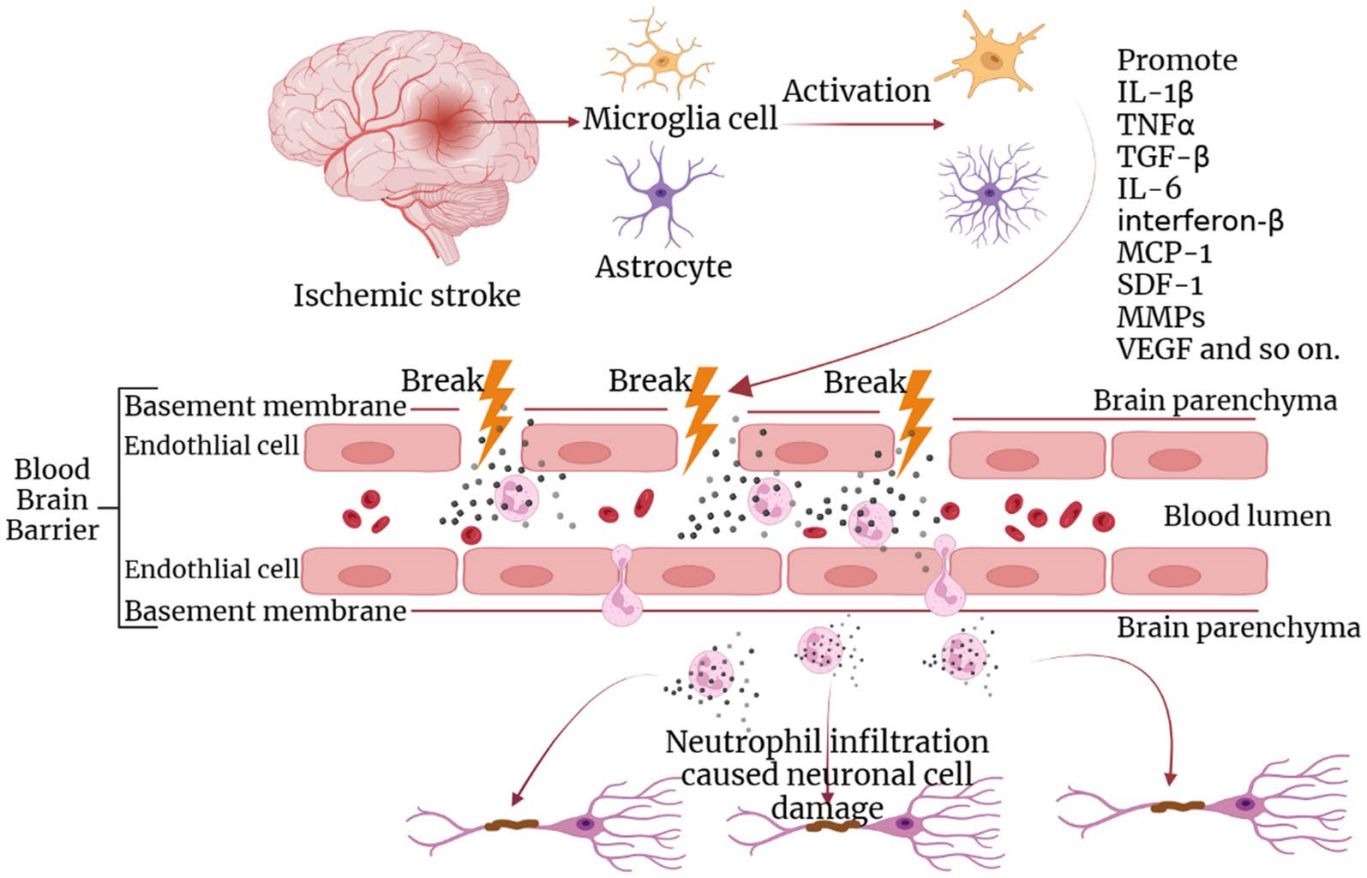
Neuroinflammatory mechanism and related factors of BBB ischemic stroke injury

 After an ischemic stroke occurs, surrounding immune cells (such as monocytes, neutrophils, T cells, and others) and microglia work together to mediate the death of neurons and the breakdown of the BBB (Fig. 4) [79]. The attack increases the BBB’s permeability, allowing immune cells and plasma proteins to enter the brain parenchyma, worsening the inflammatory process [72]. Microglia and astrocytes activation following the ischemia leads to the production of cytokines in ischemic brain tissue, and these cytokines and matrix metal proteinases (MMPs) are critical mediators in BBB injury during ischemic stroke. They cause an increase in adhesion molecules and inflammatory blood cells, primarily neutrophils, which infiltrate through the damaged BBB [80]. Interleukin-1β (IL-1β) induces pericellular secretion of matrix metalloproteinase-9 (MMP-9) through the NF-κB signaling pathway, which damages the BBB’s integrity [81, 82]. MMP-9 is involved in BBB injury pathogenesis through the potential NOTCH3/NF-κB signaling pathway. The mitogen-activated protein kinase (MAPK) signaling pathway also plays a crucial role in ischemic strokes [83–85]. P38 MAPK regulates the synthesis of occludin proteins in the BBB structure [86]. Chemokines mediate secondary brain injury by activating MAPK-related signaling pathways, making targeting and restraining the p38 MAPK pathway vital to protect the BBB’s structural integrity [87]. The Wnt/β-catenin signaling pathway plays a critical role in the formation, maintenance, and development of the BBB [4, 88–92]. Activation of this pathway leads to an increase in the expression of several Wnt ligands such as Wnt-1, Wnt-3a, and Wnt-5 A, as well as β-catenin protein [93]. The Wnt/β-catenin signaling pathway is involved in the regulation of central nervous system angiogenesis and the expression of BBB-specific transporter molecules, promoting the formation of capillary TJ proteins [94–96]. Moreover, Wnt5a can regulate endothelial cell survival, proliferation, and gene expression [97, 98]. The Wnt7a/7b ligand and Wnt/β-catenin signaling pathways drive angiogenesis in the brain and BBB generation [99]. However, during ischemic stroke attack, the expression of Wnt-3a and β-catenin is down-regulated, leading to BBB injury [93, 100]. Recent studies have demonstrated that microRNAs (miRNAs) play a crucial role in regulating changes in gene expression in brain microvascular endothelial cells associated with inflammation [101].

#### Major injury-related cytokines

interleukin-1β (IL-1β), tumor necrosis factor α (TNF- α), IL-6, IL-10, interferon β (IFN-β) and transforming growth factor β (TGF-β)), chemokines (e.g., Monocyte chemoattracted-protein 1 (MCP-1/CCL2), MIP-1α (CCL3) and SDF-1 (CXCL12)), MMPs, and vascular endothelial growth factor (VEGF).

Apart from the immune neuroinflammatory response, oxidative stress also plays a role in cerebral ischemia-reperfusion injury. These two factors interact to mediate neuron and BBB damage during ischemic stroke and subsequent hemorrhagic transformation (HT) [102]. Under physiological conditions, the oxidative stress process involves a series of peroxides and superoxides, including reactive oxygen species (ROS), reactive nitrogen species (RNS), and other reactive intermediates. These act as important regulators in the transmission of redox reaction signals. However, during ischemic stroke or other ischemia-hypoxia injuries, the physiological balance mechanism between ROS/RNS production and elimination is disrupted, leading to oxidative/nitrosative stress and persistent oxidative damage [103]. This disruption also affects electron transport chains and mitochondrial respiration, disrupting mitochondrial dynamics and ATP synthesis [104], which can injure neurons, activate the apoptosis pathway, and further lead to oxidative damage of BBB endothelial cells [105]. Reactive oxygen species (ROS) are key mediators of BBB dysfunction during oxidative stress, regulating TJ proteins and the cytoskeleton of brain endothelial cells [72]. They participate in oxidative damage, regulate TJ modification, and activate inflammatory factors, and thus play a crucial role in the various mechanisms of BBB damage, including the kinin system, excitatory toxicity from toxic glutamate efflux, neutrophil recruitment, mitochondrial changes, and macrophage/microglial activation [106, 107]. Glutathione (GSH) is another important participant in the REDOX process, and its oxidation to glutathione disulfide (GSSH) is a critical step. Interference with this REDOX metabolism may impair barrier homeostasis and produce oxidative stress [108, 109].

As mentioned earlier, when an ischemic stroke occurs in the brain, a cascade of inflammatory mediators (including cytokines, chemokines, and growth factors) is released into the damaged tissues [110, 111]. These inflammatory mediators can cause the release of MMPs, which can break down the TJ proteins and compromise the integrity of the BBB [112, 113]. TJ proteins, such as the claudin family, occludin, Zona occludens 1 (ZO-1), and tricellulin, have been identified as being related to the neuro-barrier [114]. The mechanism of BBB injury after an ischemic stroke lies in the early up-regulation of endocytosis in endothelial cells and the later remodeling of tightly connected complexes [115]. Increasing evidence suggests that the primary mechanism behind BBB leakage after a stroke is the breakdown of the TJ complex. Integrin, which influences cell adhesion, migration, and survival, has been found to have a significant impact on these processes [116]. Laminin is an ECM protein that is widely expressed in the CNS and can interact with both integrin and non-integrin receptors [117]. Numerous studies have explored the relationship between integrin and BBB permeability. After an ischemic stroke, the upregulation of integrin αvβ3 can promote angiogenesis and functional recovery [118]. Additionally, the induction of integrin α5β1 and its downstream signaling pathway play a crucial role in the pathology of ischemic stroke and cerebral hypoxia [119]. However, there is still some controversy over the expression and specific roles of laminin and various integrins during ischemic stroke [117].

Furthermore, reperfusion-induced hemorrhagic transformation is a common complication after ischemic stroke. During the initial period, leukocyte-derived MMP-9 and brain-derived MMP-2 are involved, and later, MMP-3 and MMP-9, angiogenesis, and vasogenic edema may occur, which can destroy NVUs and exacerbate the destruction of the BBB [120, 121].

### Treatment approaches

Currently, recombinant tissue-type plasminogen activator (r-tPA) thrombolytic therapy remains the most effective treatment for ischemic stroke. However, due to the narrow time window of 4–6 h after stroke onset, only a small number of patients can receive efficient thrombolytic therapy. This limited time frame poses a challenge for stroke treatment [122], and the risk of hemorrhagic transformation after application further restricts its clinical application [123].

### Improve cerebral edema

Early surgical decompression is a significant factor in improving the prognosis of cerebral edema caused by an ischemic stroke. Although therapeutic hypothermia is a potential treatment option, it remains unproven, and conservative drugs have limited efficacy in anti-edema treatment [124]. Targeting caspase-1 has been shown to reduce cerebral edema and the incidence of hemorrhagic transformation (HT) during acute stroke [125]. Caspase-1 is a family of cysteine proteases that mediate pyroptosis [126]. In acute stroke, caspase-1 is upregulated and has been shown to mediate BBB disruption [127].

### Regulates immune and inflammatory responses

Regulating immune and inflammatory responses is crucial in treating ischemic stroke. Immune cells can help eliminate necrotic tissue and promote neuron recovery, but they also release inflammatory factors that aggravate breakdown of the BBB, especially during later reperfusion [128]. Targeting immune cells in BBB disruption is a promising strategy to improve stroke prognosis and existing treatments [129]. Currently, immunoregulatory therapies are being developed to reduce pro-inflammatory cytokines, MMPs, and infiltrating leukocytes to maintain BBB homeostasis, although there are no proven clinical applications for immunoregulation yet. Ischemic stroke triggers a serious neuroinflammatory response [128, 129], which can harm neurons by releasing cytokines, chemokines, and oxidative stress-related factors. Therefore, taking corresponding measures to suppress the immune response and the occurrence and development of inflammatory processes during cerebral ischemia may be a promising target for developing new therapeutic strategies [130]. Several methods are being studied to inhibit MMPs, which mediate TJ destruction and protect the BBB from ischemic injury. Physical methods like hyperbaric oxygen, hypothermia, and drugs like isoflurane and hydrogen sulfide, or non-invasive vagus nerve stimulation, can all be used to achieve this goal [131, 132]. Activated astrocytes play an important role in a series of inflammatory reactions after an ischemic stroke. Li [133] reviewed the possibility of targeting multiple reactive astrocytes to protect the BBB and maintain brain homeostasis. Qu [134] found that gallic acid (GA) can alter microglia polarization to reduce BBB injury induced by cerebral ischemia/reperfusion with beneficial consequences.

### Eliminate oxidative stress

Mitochondrial dynamics, which include ROS generation, autophagy, and cell apoptosis, are closely linked to the pathophysiology of ischemic stroke. These dynamics also affect the body’s energy metabolism. Some researchers believe that inhibiting excessive mitochondrial division and restoring the balance of mitochondrial dynamics could be a novel approach to treating ischemic stroke [135]. Certain natural polyphenols act as powerful antioxidants that inhibit ROS production, scavenge free radicals, and improve BBB function [136]. Additionally, intravenous administration of the endogenous peptide apelin-13 can significantly reduce BBB permeability and vasogenic edema by targeting oxidative stress during ischemia-reperfusion.

### Reconstruct the BBB structural components and regulate the signaling pathway

Reconstructing the BBB is considered a promising treatment option for ischemic stroke. Kadir RRA et al. [137] established an in vitro BBB model through cell co-culture and demonstrated that overgrown endothelial cells (OECs) can effectively migrate to the injured site and restore BBB integrity. OEC-based cell therapy can also reduce oxidative stress and apoptosis of cerebral microvascular endothelial cells after ischemic stroke injury. M. Alwjwaj [138] later found that OEC acts as a therapeutic agent to prevent ischemia by specifically inhibiting NOX2, a major source of vascular oxidative stress, and thereby reducing oxidative stress. Zeng et al. [139] conducted in vivo and in vitro experiments and found that the DNA methyltransferase inhibitor Zebularine can reduce the production of pro-inflammatory factors and improve brain edema and nerve function by increasing the expression of ZO-1 and vascular endothelial (VE)-cadherin [131, 132]. Nilles KL reviewed [140] efforts to optimize the success rate of stroke drug conversion across the BBB by targeting BBB transporters. Song et al. found that selective loss of Nhe1 (a Ph-sensitive Na + /H + exchanger 1) in astrocytes can increase the expression of Wnt7a/7b protein and preserve Wnt/β-catenin signal in endothelial cells, improve angiogenic repair, cerebral blood perfusion, and maintain the integrity of the BBB after ischemic stroke [141]. Recent studies have also been conducted to synthesize specific Wnt activators, such as Wnt7a, through genetic engineering to protect the BBB [142]. Studies suggest that Fluoxetine can increase the expression of Claudin-5 and its effect on Wnt signaling may help restore BBB function while promoting neurogenesis [143, 144]. Lithium can lower the expression of MMP-9, increase the activity of Wnt/β-catenin signaling, and preserve the integrity of the BBB by raising the TJ protein levels (Claudin-5 and ZO-1) [145]. Laksitorini et al. reported that activation of Wnt/β-catenin by LiCl (GSK3 inhibitor) or Wnt3a can improve brain endothelial barrier function in the BBB cell culture model in vitro [144]. Liu et al. demonstrated that the glucagon-like peptide-1 receptor (GLP-1R) agonist exendin-4 (EX-4) inhibits ROS production and MMP-9 activation by activating the Wnt/β-catenin signaling pathway in a rat model of temporary middle cerebral artery occlusion. Therefore, GLP-1R agonists can be potential protective agents to reduce the risk of HT after r-tPA treatment [146]. Golgi matrix protein 130 (GM130) [113]can up-regulate the inhibition of the autophagy-lysosome pathway, maintain appropriate autophagy to prevent the damage of tight connections, maintain BBB function, and reduce brain parenchymal injury. Exosomes isolated from the venous serum of healthy individuals can also play a neuroprotective role against experimental stroke by inhibiting endothelial cell apoptosis and BBB breakdown mediated by autophagy [147, 148]. This approach shows promise as a potential treatment for ischemic stroke through the BBB in the future.

### The application of novel nanomaterials

The BBB is a significant obstacle to drug delivery to the brain parenchyma [149]. However, this barrier can be overcome by opening or crossing the endothelial barrier, and specific sites can be targeted to protect corresponding organs [150]. Therefore, there is growing interest in studying the delivery of neuroprotective agents to enable drugs to cross the BBB and reach their target sites in the brain parenchyma. Nanoparticles such as liposomes have been used for drug delivery, as they can minimize chemical degradation, improve drug permeability, and achieve the necessary blood drug concentration at the site of action [151–153]. For instance, cerium (Ce)-doped Linde Type A (LTA) zeolite-based nanomaterials (Ce/Zeo-NMs) have been shown to use their unique adsorption capacity and simulated catalytic activity to remove reactive oxygen species, enhance the integrity of the BBB, inhibit the activation of inflammatory cells, and reduce neurovascular dysfunction [154]. Betulinic acid, one of the most potent stroke antioxidants, can be delivered by naturally compound-derived nanoparticles (NPs) to improve stroke recovery [155]. Moreover, nanomaterials coated with drugs, such as uPA-loaded black phosphorus nanosheets (BPNs), can effectively deliver uPA across the BBB to dissolve thrombi and remove ROS [156]. Brain-targeting bionic nanomaterials (RR@SABNPs) can significantly prolong the half-life of salvianolic acid B (SAB), deliver SAB to the ischemic brain, and demonstrate a good therapeutic effect in model mice [157]. The nanogel system is a promising drug delivery platform that could be used therapeutically as a more stable and superior option for crossing the BBB [140, 158]. Invasive techniques such as ultrasound drug delivery, craniotomy drug delivery, and other methods are related to brain drug delivery strategies targeting intracranial diseases [155, 159].

### Chinese medicine treatment

Currently, it has been discovered that some traditional Chinese medicines or prescriptions can protect against ischemic stroke through various mechanisms. The Qishen Yiqi formula [160] protects against ischemic stroke through a synergistic lysosomal/inflammatory mechanism. Certain Chinese herbs [161] can enhance their thrombolytic ability and reduce the risk of hemorrhagic transformation as tPA adjuncts. Astragaloside IV (AST IV) and total saponins of notoginseng (PNS) are the main effective components of Astragaloside IV and notoginseng for treating ischemic stroke, respectively. When combined with borneol, these components can promote their delivery by down-regulating the expression of effector transporters and up-regulating the expression of uptake transporters, thereby enhancing the protective effect of ischemia-reperfusion and maintaining BBB integrity [162]. Moreover, other studies have shown that acupuncture can activate the inherent antioxidant enzyme system, inhibit the overgeneration of reactive oxygen species, reduce the potential of oxidative stress caused by cerebral ischemia, and play a neuroprotective role [163, 164].

### Other methods

Clinical trials have suggested that stem cell therapy holds promise in improving the sequelae of patients with acute ischemic stroke, although further verification is still needed. Neural stem cell transplantation, which can replace damaged neural cells and maintain the BBB through the bystander effect, is a potential therapy for neurovascular diseases [165, 166]. In recent years, microRNA has also received significant research attention due to its negative impact on BBB injury after ischemic stroke. Intervening with microRNA can control BBB permeability through various mechanisms [131, 167]. Further research indicates that microRNA could serve as a promising BBB modulator for ischemic brain injury. Another study found that the activation of vascular endothelial growth factor (VEGF) can strengthen the vasculature in stroke patients and prevent the progression of secondary brain damage. Furthermore, administering VEGF after a stroke can repair the BBB and reduce secondary brain edema damage [168].

**Fig. 5:**
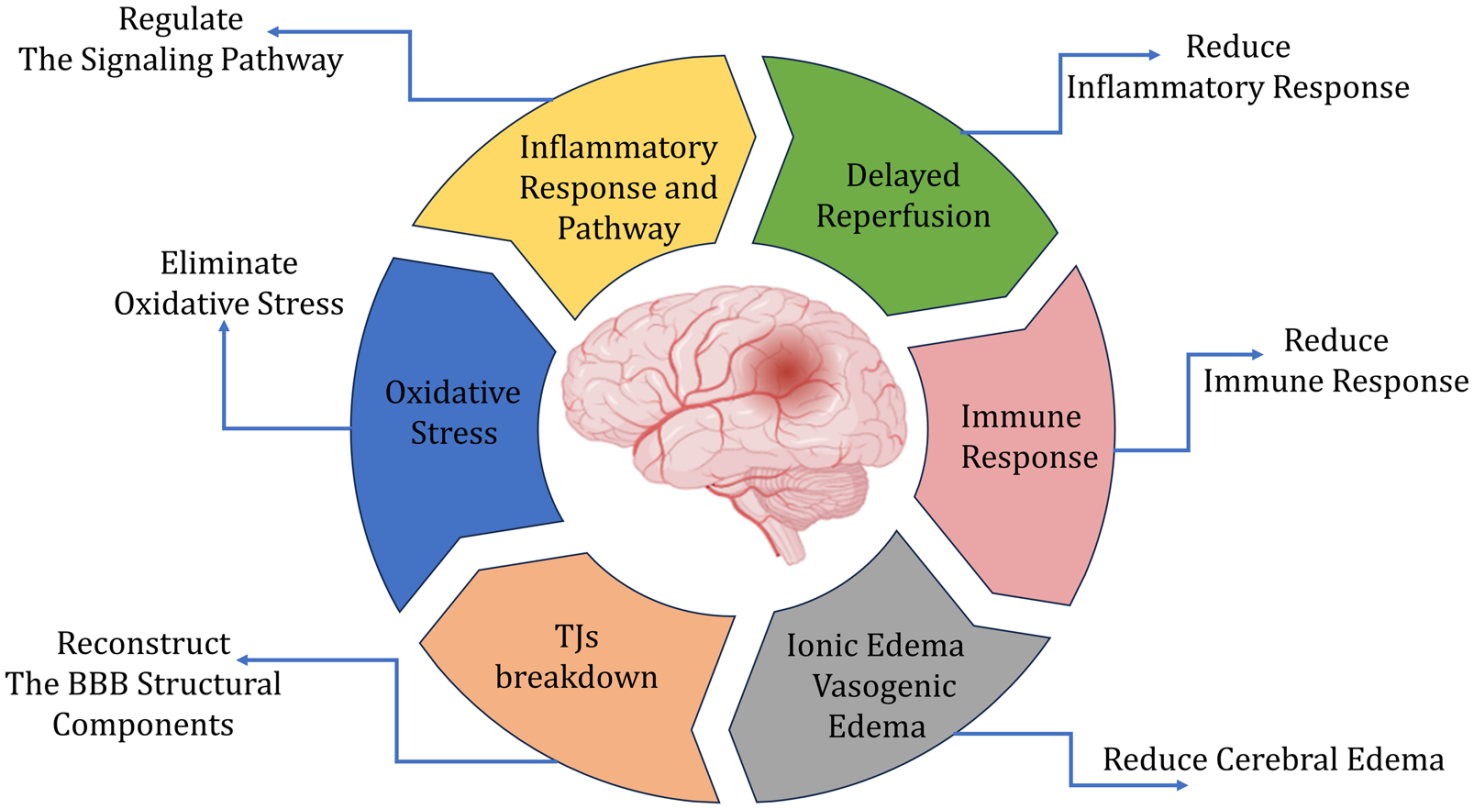
Graphic abstract of this article

What’s more, the application of single-cell sequencing in the analysis and collection of genetic information related to clinical diseases is increasingly prevalent. Among them, RNA sequencing (RNA-Seq) is widely used and has gradually become an indispensable tool for analyzing differential gene expression. Based on this, a large number of RNA sequences have also been introduced into BBB research in recent years. The earliest genomFIGHic analysis of BBB components using RNA-Seq identified that genes related to tight junctions, signaling molecules, and some other molecular genes are highly enriched at the BBB, indicating the involvement of Wnt and RXRalpha in the signaling cascade regulation of this barrier [169]. David and his team summarized relevant scientific experience to establish guidelines that promote RNA-seq research on the BBB [170]. He and his team captured corresponding vascular cells using transgenic reporter mice, thus constructing a database of mouse brain and lung vascular and vascular-related cell types. This database will provide a solid foundation for vascular development and disease research [171]. There are also some other relevant studies that compare the transcriptional gene expression differences between human and mouse brain micro-vessels, revealing their potential impact on drug delivery and diseases. After all, increasing the understanding of gene expression differences between the mouse and human brain vascular systems is crucial to assessing the potential limitations of mouse models for studying brain vasculature and the BBB development and diseases [172]. Furthermore, through single-cell sequencing and gene expression analysis of differential results, it is possible to guide experimental research in the search for disease target factors. Recently, a study using RNA-Seq has provided a detailed comparative characterization of the molecular profiles of endothelial cells in normal human brain tissue and glioblastoma, aiming to provide valuable information for drug delivery across the BBB and intra-tumoral distribution [173]. Other studies have used RNA-seq to explore potential targets and signaling pathways related to BBB dysfunction induced by A1 astrocytes. It is inferred that blocking the transformation of C3d+/GFAP + A1 astrocytes may alleviate BBB disruption in mice after ischemic stroke [174] (Fig. 5).

## Conclusion and expectation

By providing a summary of our review, we aim to offer new insights for enhancing the prognosis of future research on the treatment of ischemic stroke by safeguarding the integrity of the BBB. We are confident that, with the collective efforts of an increasing number of researchers, more effective and innovative approaches will be developed and applied in clinical settings to alleviate the social and economic burden that this disease places on families.

## Data Availability

Not applicable.

## Abbreviations

BBB: Blood brain barrier
ATP: Adenosine triphosphate
IL-1β: Interleukin-1β
MAPK: Mitogen-activated protein kinase
HT: Hemorrhagic transformation
ROS: Reactive oxygen species
RNS: Reactive nitrogen species
GSH: Glutathione
GSSH: Glutathione disulfide
REDOX: Reduction–oxidation
r-tPA: Recombinant tissue-type plasminogen activator
GA: Gallic acid
OECs: Overgrown endothelial cells
NOX2: NADPH oxidase 2
GLP-1R: Glucagon-like peptide-1 receptor
GM130: Golgi matrix protein 130
Ce/Zeo-NMs cerium: (Ce)-doped Linde Type A (LTA) zeolite-based nanomaterials
NPs: Nanoparticles
uPA: Urokinase-type plasminogen activator
BPNs: Black phosphorus nanosheets
SAB: Salvianolic acid B
AST IV: Astragaloside IV
PNS: Saponins of notoginseng
VEGF: Vascular endothelial growth factor
